# Role of Somatostatin Receptor-2 in Gentamicin-Induced Auditory Hair Cell Loss in the Mammalian Inner Ear

**DOI:** 10.1371/journal.pone.0108146

**Published:** 2014-09-30

**Authors:** Yves Brand, Vesna Radojevic, Michael Sung, Eric Wei, Cristian Setz, Andrea Glutz, Katharina Leitmeyer, Daniel Bodmer

**Affiliations:** Department of Biomedicine, University Hospital Basel, Basel, Switzerland and Clinic for Otolaryngology, Head and Neck Surgery, University Hospital Basel, Basel, Switzerland; Temple University, United States of America

## Abstract

Hair cells and spiral ganglion neurons of the mammalian auditory system do not regenerate, and their loss leads to irreversible hearing loss. Aminoglycosides induce auditory hair cell death *in vitro*, and evidence suggests that phosphatidylinositol-3-kinase/Akt signaling opposes gentamicin toxicity via its downstream target, the protein kinase Akt. We previously demonstrated that somatostatin—a peptide with hormone/neurotransmitter properties—can protect hair cells from gentamicin-induced hair cell death *in vitro*, and that somatostatin receptors are expressed in the mammalian inner ear. However, it remains unknown how this protective effect is mediated. In the present study, we show a highly significant protective effect of octreotide (a drug that mimics and is more potent than somatostatin) on gentamicin-induced hair cell death, and increased Akt phosphorylation in octreotide-treated organ of Corti explants *in vitro*. Moreover, we demonstrate that somatostatin receptor-1 knockout mice overexpress somatostatin receptor-2 in the organ of Corti, and are less susceptible to gentamicin-induced hair cell loss than wild-type or somatostatin-1/somatostatin-2 double-knockout mice. Finally, we show that octreotide affects auditory hair cells, enhances spiral ganglion neurite number, and decreases spiral ganglion neurite length.

## Introduction

Sensorineural hearing loss is linked to degeneration and death of auditory hair cells (HCs) and their associated spiral ganglion neurons (SGNs), which is irreversible in mammals. Therefore, developing therapeutic strategies for hearing loss prevention requires a better understanding of the survival pathways and molecular events involved in auditory epithelium protection. Until recently, auditory HC and SGN damage has been considered an inevitable consequence of age, genetic conditions, exposure to ototoxic drugs, or certain environmental stimuli. However, several recent studies using *in vitro* aminoglycoside-induced HC death as a model have discovered some of the critical intracellular events that mediate HC damage [1], [2], [3], [4]. Aminoglycoside exposure sets into motion a series of cellular and biochemical alterations in the HCs. Reactive oxygen species have been detected *in vitro* shortly after exposure to aminoglycosides [5]. It has also been demonstrated that small GTPases (e.g., Ras and Rho/Rac/Cdc42) and the c-jun-N-terminal kinase (JNK) signaling pathway are activated in aminoglycoside-exposed cells [6], [7], [8], [9]. Finally, caspases are activated and HCs undergo apoptotic cell death after prolonged aminoglycoside exposure [10], [11]. Interestingly, phoshatidylinositol-3-kinase (PI3K) signaling reportedly mediates HC survival and opposes gentamicin toxicity via its downstream target, the protein kinase Akt [12]. Despite the progress made towards understanding the processes involved in auditory HC death and survival, there is still no available cure for individuals with sensorineural hearing loss; only auditory prosthesis (e.g., hearing aids or cochlear implant) can offer some help to individuals with hearing loss.

The regulatory peptide somatostatin (SST) acts on a wide array of target tissues to modulate neurotransmission, cell secretion, and cell proliferation [13], [14]. SST actions are mediated by five subtypes of G protein-coupled receptors (SST receptors 1–5) that are encoded by separate genes [14], [15]. These SST receptors modulate several intracellular signaling transduction pathways, including the Mek/Erk, PI3K-Akt, and p38 pathways [16]. Octreotide acetate—a long-acting octapeptide with SST-mimicking pharmacologic actions—inhibits growth hormone, glucagon, and insulin even more potently than SST does. Studies in mice show that SST and its receptors appear to play an important role in cell death. In a retina ischemia model, SST receptor-2 activation protected retinal neurons from damage [17].

Our group has previously studied the somatostatinergic system in the mammalian inner ear [18], [19], [20]. We demonstrated that SST receptor-1 and -2 are specifically expressed in the outer and inner HCs of the organ of Corti (OC), and in defined supporting cells. Interestingly, SST itself was not expressed in the mammalian cochlea. Most importantly, in *in vitro* studies, we found improved auditory HC survival in OC explants treated with gentamicin and SST compared to in explants treated only with gentamicin. However, the intracellular events mediating these effects remain unknown, and the effects on SGN were not evaluated.

In the current *in vitro* study, we examined whether octreotide could protect mammalian auditory HCs from gentamicin-induced HC death. We also investigated whether this drug increased Akt phosphorylation in the OC. Furthermore, we examined SST receptor-1 knockout mice and confirmed their overexpression of SST receptor-2 in the OC, and evaluated their susceptibility to gentamicin-induced HC loss compared to that of wild-type and SST-1 receptor/SST receptor-2 double-knockout mice. Finally, we evaluated the effects of octreotide on SGN survival and neurite outgrowth.

## Material and Methods

### Animal procedures

All animal procedures were performed in Basel, Switzerland, following an animal research protocol approved by the Committee on the Ethics of Animal Experiments of Basel (Kantonales Veterinäramt Basel, Permit Number: 2263), in accordance with the European Communities Council Directive of 24 November 1986 (86/609/EEC). Animals were sacrificed prior to all tissue extractions. The procedures used to generate homozygous SST receptor 1 knockout (SST receptor-1^−/−^)/C57BL6J mice and homozygous SST receptor-2 knockout (SST receptor-2^−/−^)/C57BL6J mice have been previously described [21], [22]. SST receptor-1^−/−^ mice were crossed with SST receptor-2^−/−^ mice to produce double-knockout mice. Age-matched wild-type mice were produced from the C57BL6J mice used to stabilize the genetic backgrounds of the knockout mice [20]. All experiments used either the above-described mice or Wistar rats (Harlan, Indianapolis, IN, USA).

### OC tissue culture

Five-day-old Wistar rat pups (Harlan, Indianapolis, IN, USA) and seven-day-old wild-type, SST receptor-1 knockout, and SST receptor-1/SST receptor-2 double-knockout mice were decapitated, and then cochlear microdissections were performed under a light microscope to isolate the OC and the spiral ganglion (SG) as described by Sobkowicz *et al*. [23]. OCs were initially incubated in cell culture media containing Dulbecco's modified Eagle's medium (DMEM) supplemented with 10% fetal calf serum (FCS), 25 mM HEPES, and 30 U/mL penicillin (Invitrogen, Carlsbad, CA, USA) at 37°C with 5% CO_2_, followed by recovery for 24 hours under these conditions.

Next, the OCs were transferred into fresh cell culture media and incubated at 37°C with 5% CO_2_ for 20 hours (mouse tissue culture) or for 48 hours with the solution exchanged once after 24 hours (rat tissue culture). HC damage was induced by incubating OCs with gentamicin (Sigma-Aldrich, St. Louis, MO, USA) in the cell culture medium: 48 hours with 50 µM gentamicin for rat tissue cultures, or 20 hours with 0.5 mM gentamicin for mouse tissue culture. Then the rat tissue OCs were pretreated for 24 hours with increasing amounts of octreotide (Novartis Pharma, Switzerland), with final concentrations of 1 µM or 5 µM in the cell culture medium. After this pretreatment, rat OCs were exposed either to 50 µM gentamicin and 1 µM octreotide, 50 µM gentamicin and 5 µM octreotide, or only 5 µM octreotide for 48 hours. Other Wistar rat OCs were not pretreated and were incubated for 48 hours either in culture medium alone (controls) or in culture medium with 50 µM gentamicin. Mouse tissue cultures were prepared similarly but without addition of octreotide to the cell culture media.

Finally, additional experiments using the Akt-inhibitor SH-6 (AG Scientific, San Diego, CA, USA) were performed. Rat OCs were transferred after recovery as described above into fresh culture media and incubated at 37°C with 5% CO_2_ for 48 hours. For the gentamicin-alone condition, explants were exposed to culture medium containing 200 µM gentamicin. A new gentamicin batch was used and gentamicin dose was adjusted to provide the same degree of HC damage as in the rat tissue experiments above. It is known that gentamicin toxicity various between different batches of gentamicin [12]. The following conditions were applied: OCs were pretreated for 24 hours with 5 µM octreotide and changed to cell culture media containing 200 µM gentamicin and 5 µM octreotide for 48 hours. OCs were pretreated for 24 hours with 5 µM octreotide and 10 µM SH-6 and changed to cell culture media containing 200 µM gentamicin, 5 µM octreotide and 10 µM SH-6 for 48 hours.

OCs were pretreated for 24 hours with 10 µM SH-6 and changed to cell culture media containing 10 µM SH-6 for 48 hours. In all conditions, the solution was exchanged once after 24 hours.

### HC count and statistical analysis

OCs were fixed in 4% paraformaldehyde, and permeabilized with 5% Triton X-100 in phosphate-buffered saline (PBS) containing 10% FCS. The OCs were then incubated with a 1∶100 dilution of Texas Red X-phalloidin (Molecular Probes, Eugene, OR, USA) for 45 minutes at room temperature. After fixation, the OCs were visualized and photographed using a fluorescence microscope (Olympus IC71, Center Valley, PA, USA).

Quantitative analysis was performed by evaluating 60 OHCs associated with 20 IHCs in a given microscopic field. Explants were randomly analyzed for the middle and basal turn, with three random microscope fields counted and averaged for each explant. These values were then averaged across the six replications of each experiment in rat OC cultures, and across the 15 replicates of each experiment in mouse OC cultures. Inner HCs and outer HCs were counted and used to assess HC survival. HC counting results were analyzed by analysis of variance (ANOVA), followed by the least-significant difference (LSD) post-hoc test (Stat View 5.0). Differences associated with *p* values of <0.05 were considered to be statistically significant. All data are presented as mean ± SD.

### Assessment of Akt Activation

To assess activation of the PIK3/Akt signaling pathway for each condition, we harvested 10 intact OCs from 5-day-old Wistar rat pups (Harlan) and incubated them overnight in cell culture media, as described above. Subsequently, OC explants were placed in cell culture media with or without 5 µg/mL octreotide (Novartis Pharma) for one hour. Explants were separately collected from the media and lysed with 150 µL T-Per Tissue Protein Extraction Reagent (Thermo Scientific, Rockford, IL, USA) containing 1× phosphatase and protease inhibitors (Roche, Indianapolis, IN, USA). The samples were centrifuged at 14,000 rpm for 10 minutes to separate the cytosolic portion from the membranous components. Supernatants containing the proteins were sonicated for 5 seconds to shear chromosomal DNA. The protein level was then determined using the bicinchoninic acid (BCA) method (Thermo Scientific, Rockford, IL, USA). The samples were diluted 1∶4 in 4× NuPAGE LDS sample buffer and 1∶10 in 10× NuPAGE reducing agent (Invitrogen Life Technologies, Green Island, New York, USA), and then heated to 95°C for 5 min, and placed on ice to cool. Next, 10 µg of each sample was separated by SDS-PAGE in a 9% acrylamide gel, and electrotransferred to a nitrocellulose membrane. The membrane was blocked with 3% TopBlock (Lubio Science, Lucerne, Switzerland) in TBS-Tween (50 mM Tris-HCL, pH 7.4; 150 mM NaCl; 0.05% Tween 20) for 1 hour at room temperature. Blots were incubated with primary antibodies in 3% TopBlock/TBS-Tween overnight at 4°C, and then incubated 1 hour with α-DyLight800-coupled α-rabbit and α-mouse antibodies (Pierce). Bands were visualized using an infrared-based laser scanner (LiCor), and blots were evaluated with rabbit anti-p-Akt and rabbit anti-total Akt (Cell Signaling Technology, Beverly, MA, USA), as well as with mouse anti-β-actin (Abcam, Cambridge, UK) as a loading control.The intensity of the bands corresponding to p-Akt were quantified. Band intensity for p-Akt was corrected for intensity of total Akt and then expressed as the percentage increase, compared with non-treated tissue. Western blotting was replicated three times with independent biological replicate. With each biological replicate, western blotting was performed twice. 10 OCs were used per individual blot. Ratio data were analyzed using the Mann–Whitney nonparametric statistical test.

### RNA extraction

The cochleas of 14-day-old and 21-day-old wild-type and SST receptor-1 knockout mouse pups were microdissected and then the OC was isolated. Then the OC was placed separately in RNAlater (Qiagen, Hombrechtikon, Switzerland). RNA was isolated using the RNAeasy Minikit (Qiagen) and an Ultra-Turrax T8 tissue homogenizer (IKA-Werke, Staufen, Germany) following the manufacturer's instructions, including DNase treatment. Isolated RNA quantity and quality were determined with a NanoDrop ND 1000 (NanoDrop Technologies, Delaware, USA). All samples had a 260/280-nm ratio of between 1.8 and 2.1.

### Real-time PCR

Total RNA (500 ng) was reverse transcribed into cDNA using the First Strand cDNA synthesis kit (Roche Applied Biosciences) following the manufacturer's instructions. The reaction was performed in an ABI Prism 7900 HT Sequence Detection System (Applied Biosystems) using Fast Start Universal SYBR Green Master (Rox; Roche Applied Biosciences Foster City, USA) and 300 nM primer per reaction. The primer sequences were as follows: SSTR2-fwd, 5′-TCTTTGCTTGGTCAAGGTGA-3′; and SSTR2-rev, 5′-TCCTGCTTACTGTCGCTCCT-3′ (Microsynth, St. Gallen, Switzerland). The reaction conditions were as follows: 95°C for 10 min, followed by 40 cycles of 95°C for 15 s and 60°C for 60 s.

### Statistical analysis of real-time PCR

The relative quantities of specifically amplified cDNA were calculated using the comparative threshold cycle method, with GAPDH used as an endogenous reference (Microsynth). Template-free and reverse-transcription-free controls were analyzed to exclude non-specific amplification and DNA contamination. One-way analyses of variance (ANOVA) followed by Student's *t*-test with post-hoc Bonferroni's correction was performed. Means were considered significant when *p*<0.05. The Origin computer program (Microcal Software, Inc., Northampton, MA) was used for statistical analysis and to generate graphs.

### Preparation of tissue culture plates for rat SGN experiments

To prepare uniformly coated 24-well cell culture plates (Costar, Corning Inc., Acton, MA, USA), wells were filled with 300 µL of 5 µg/mL poly-L-lysine (PLL) (Sigma-Aldrich) in DMEM (Gibco by Invitrogen, Carlsbad, USA) and incubated at 37°C for 1 hour. The wells were then washed twice with PBS. Next, these prepared wells were filled with 170 µL primary attachment medium, containing DMEM (Gibco), 10% fetal bovine serum (Sigma-Aldrich), 25 mM HEPES buffer (Gibco), and 300 U/mL penicillin (Sigma-Aldrich).

### SGN cell culture

The spiral lamina containing the SG was carefully separated from the modiolus, and immediately transferred into primary cell culture medium. It was then cut into equal 300- to 500-µm portions and transferred to the prepared culture plates. The explants were first incubated for 24 h at 37°C in primary attachment medium, after which the culture medium was changed to serum-free maintenance media, comprised of DMEM (Gibco), 25 mM Hepes-Buffer (Gibco), 6 mg/mL glucose (Gibco), 300 U/mL penicillin (Sigma-Aldrich), and 30 µg/mL N_2_ supplement (Gibco). The maintenance medium was supplemented with 10 ng/mL recombinant BDNF for trophic support of SG neuron survival and optimization of neurite outgrowth (R&D Systems, Minneapolis, MN, USA). Cultures were kept in a humidified incubator at 5% CO_2_ and 37°C for 72 h. In experimental cultures, 1, 10, or 20 µM octreotide (Novartis Pharma) was added to the maintenance media. Maintenance media without octreotide served as a control.

### SGN Immunohistochemistry

Explants were fixed with 4% paraformaldehyde for 20 min at room temperature and washed twice with PBS (Gibco). Then they were permeabilized with 5% triton X-100 (Sigma-Aldrich) for 10 min and washed twice with PBS, and non-specific antibody binding was blocked with 5% donkey serum (Sigma-Aldrich). Neurites were labeled for neurofilament using a mouse polyclonal 200-kDa anti-neurofilament primary antibody (1∶400; Sigma-Aldrich). After overnight incubation at 4°C with primary antibody, and two washes with PBS, the neurites were visualized by 2.5 h of incubation with fluorescein isothiocyanate (FITC)-conjugated secondary antibodies (1∶100; Jackson Immunoresearch, West Grove, PA, USA) against the species of the primary antibody. Staining specificity was confirmed by a series of negative control stainings without primary antibodies.

### SGN data analysis

Digital images were obtained using a fluorescence microscope (Olympus IX71, Center Valley, PA, USA) equipped with appropriate excitation and emission filters for FITC. For publication in this manuscript, the images were optimized to achieve uniform brightness and contrast using Adobe Photoshop (Adobe Systems Inc., San Jose, CA, USA). Neurite outgrowth from the SG was evaluated by measuring the number and lengths of the processes. Images of the immunostained cultures were analyzed using NIH ImageJ software (NIH, Bethesda, MD, USA). Each neurite was traced, and the neurite numbers and average lengths per explant were analyzed using a one-way analysis of variance (ANOVA) followed by a Tukey LSD post-hoc test. Data presented in the text and figures are means and standard deviations. Results were considered significant when the likelihood for a type 1 error was less than 5% (*p*<0.05). Twenty SG explants were analyzed per experimental condition.

## Results

### Octreotide has no toxic effect on HCs, and highly protects HCs from gentamicin-induced HC damage *in vitro*

**Figure 1:**
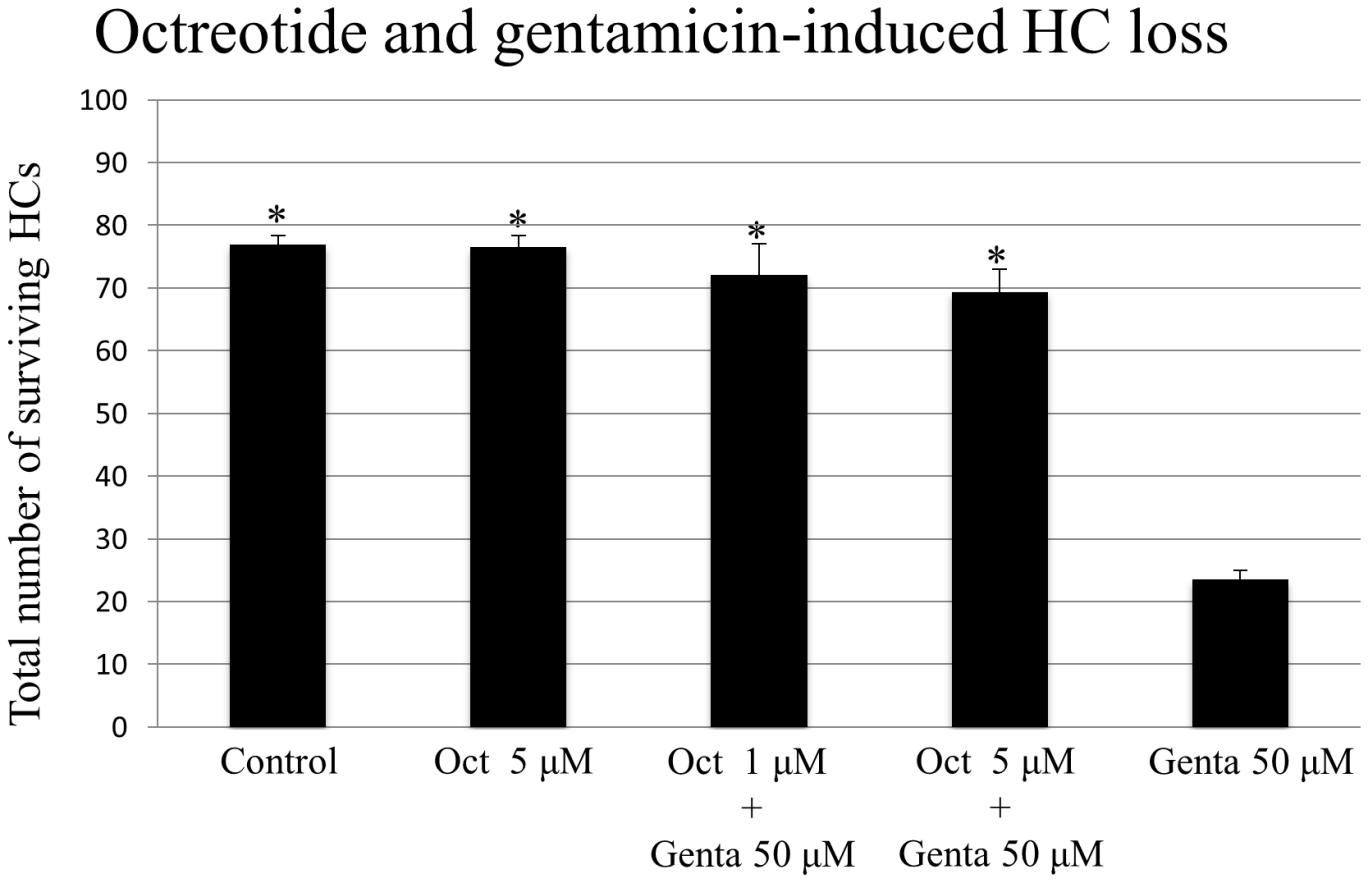
Quantitative analysis of surviving HCs under different experimental conditions in the basal and middle cochlear turns. HC survival was significantly higher in groups treated with octreotide and gentamicin compared to with gentamicin treatment alone (*p*<0.01 octreotide 1 µM, *p*<0.01 octreotide 5 µM). No toxic effects were observed in OCs incubated with octreotide without gentamicin or with culture medium only (control). Asterisks indicate significant difference from treatment with gentamicin only (p<0.01 for all conditions). Data are expressed as the mean number of surviving HCs per 20 inner HCs. Vertical lines represent one standard deviation. *n* = 6 for each experimental condition.

**Figure 2:**
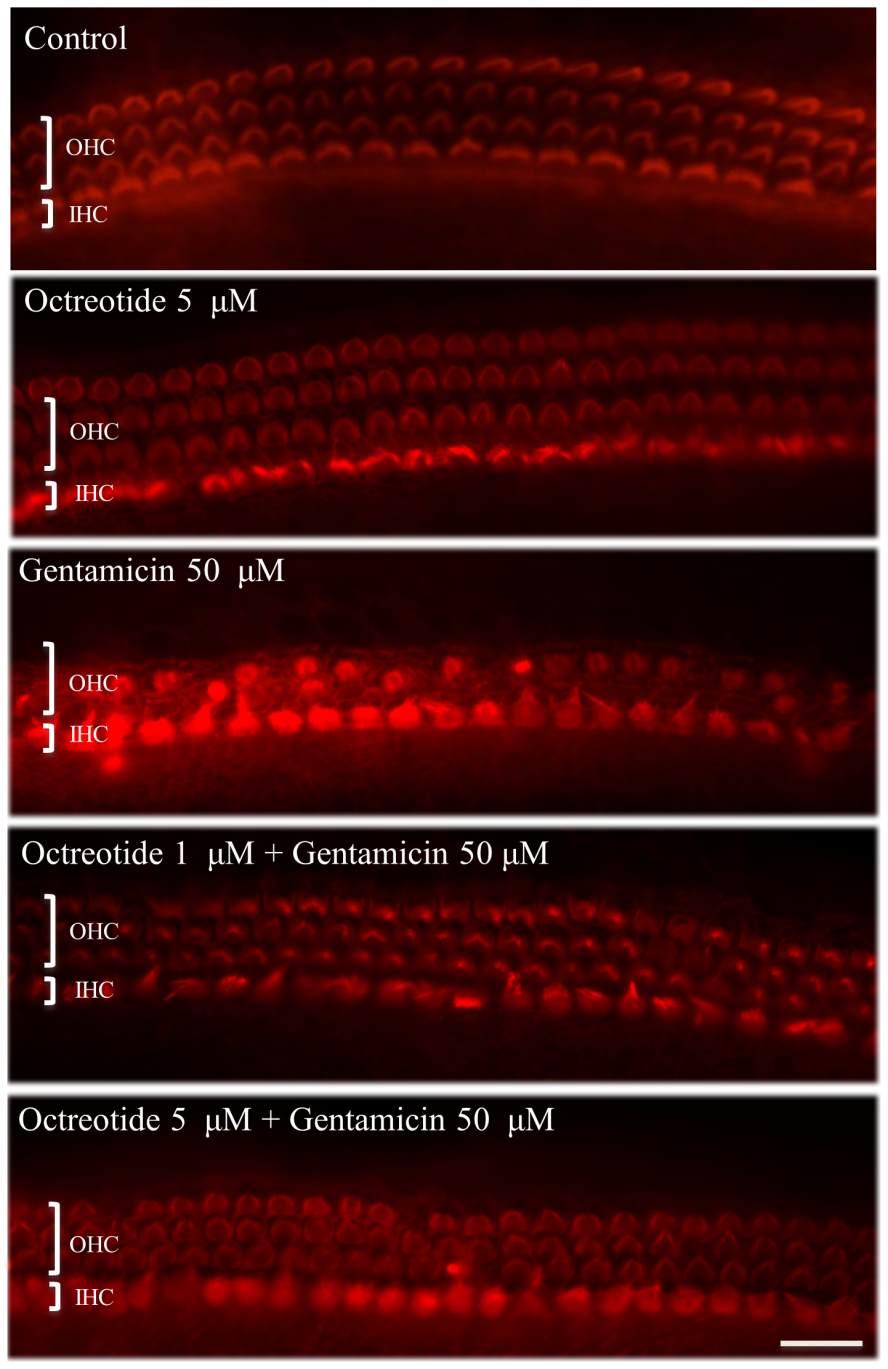
Effects of octreotide on gentamicin-induced HC damage. Three orderly rows of outer HCs (OHC) and a single row of inner HCs (IHC) were observed in control OCs and in OCs exposed to 5 µM octreotide without gentamicin. Comparatively, OCs cultured with gentamicin showed significant loss of HCs. Addition of increasing concentrations of octreotide to gentamicin-treated OCs resulted in significantly decreased HC loss compared to in those treated only with gentamicin. Scale bar  = 20 µm.

The number of surviving HCs in rat OCs cultured with the highest octreotide dosage used in this study (5 µM) for 72 hours did not differ compared to in those cultured without octreotide (Fig. 1), thus excluding a toxic effect of octreotide. Untreated control OCs and those treated with octreotide each showed three orderly rows of outer HCs and a single row of inner HCs (Fig. 2). As expected, gentamicin treatment led to HC loss (Fig. 2). Treatment with both gentamicin (50 µM) and octreotide (1 µM and 5 µM) significantly increased HC survival (Fig. 1; ANOVA, *p*<0.01 for all conditions)

### Octreotide increases Akt phosphorylation *in vitro*

**Figure 3:**
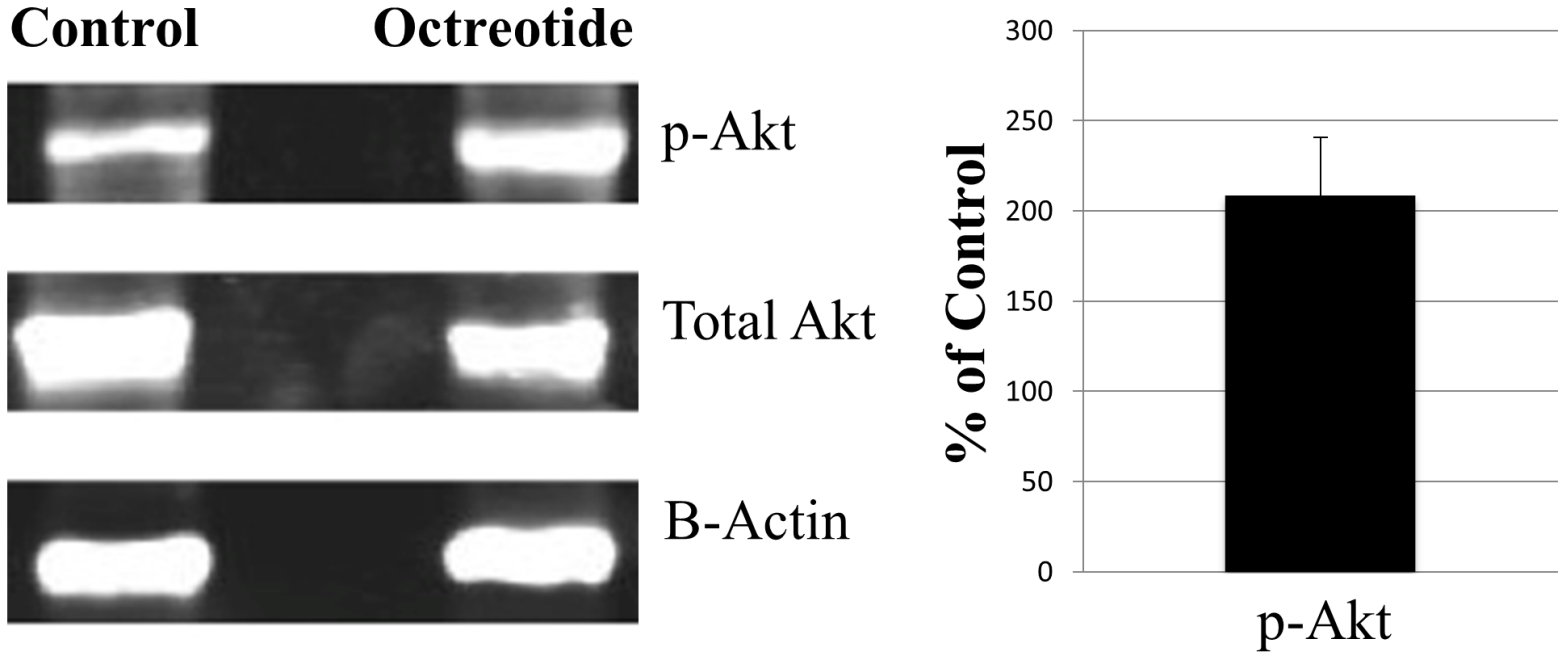
Representative western blots of p- Akt, total Akt, and β-Actin. OCs were exposed for 1 hour to either control media (Control) or media containing 5 µM octreotide. P-Akt levels were normalized against total Akt. Octreotide treated levels are expressed as % of control values. P-Akt levels were significantly increased by octreotide treatment (p<0.05). Bars show the mean ± one standard deviation of 3 independent experiments. In each experiment 10 OCs from 5 animals were used.

Western blotting revealed specific activation of Akt in OCs treated with octreotide *in vitro*. Blots using anti-p-Akt revealed strongly increased Akt activation after a 1-hour exposure to 5 µM octreotide, with both total Akt and β-actin used as references. Using total Akt as internal control, normalized p-Akt was expressed as % of control. In three replicates, the relative intensity of p-Akt was significantly increased in octreotide treated OCs compared to OCs in culture media only (Fig. 3; Mann-Whitney test, p<0.05).

### The Akt inhibitor SH-6 reverses the protective effect of octreotide on gentamicin-induced HC damgage *in vitro*

**Figure 4:**
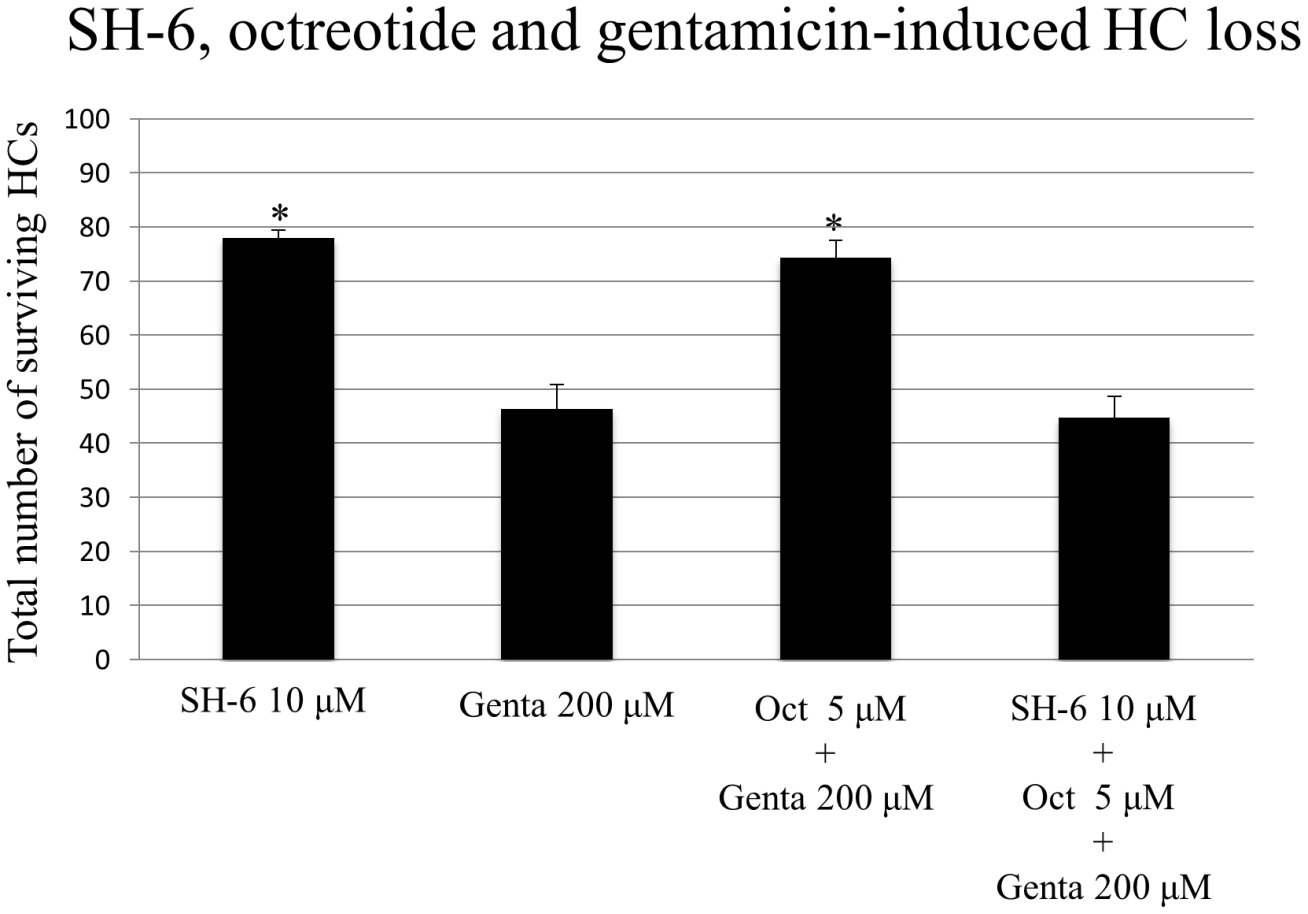
Quantitative analysis of surviving HCs under different experimental conditions in the basal and middle cochlear turns. No toxic effects were observed in OCs incubated with the AKT inhibitor SH-6 at 10 µM. HC survival was significantly higher in groups treated with octreotide and gentamicin compared to gentamicin treatment alone or treatment with SH-6, octreotide and gentamicin. Asterisks indicate significant difference from treatment with gentamicin only (p<0.01 for all conditions). Data are expressed as the mean number of surviving HCs per 20 inner HCs. Vertical lines represent one standard deviation. *n* = 6 for each experimental condition.

**Figure 5:**
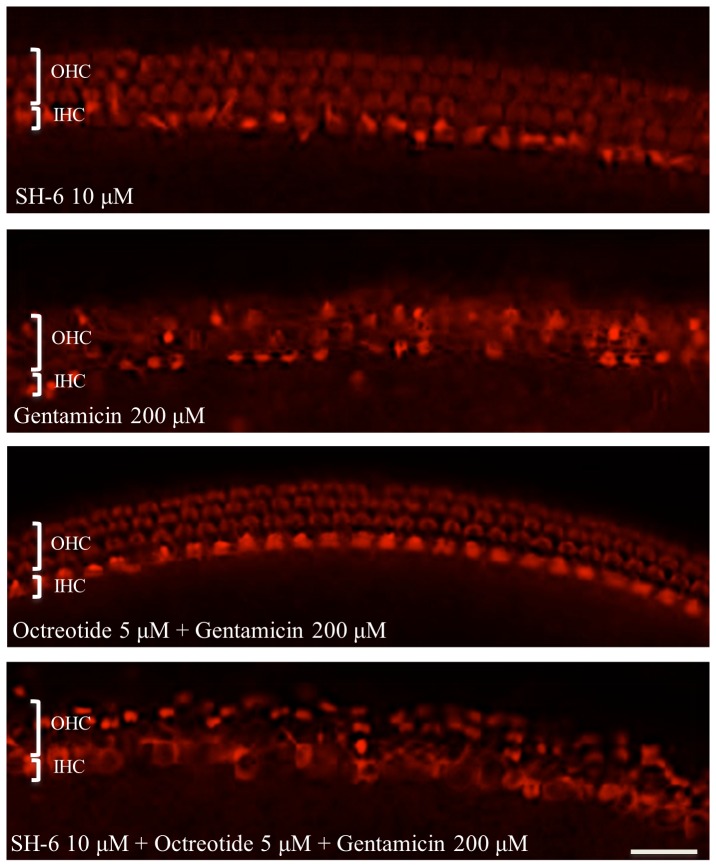
Effects of octreotide and the Akt inhibitor SH-6 on gentamicin-induced HC damage. Three orderly rows of outer HCs (OHC) and a single row of inner HCs (IHC) were observed in OCs exposed to 10 µM SH-6. Comparatively, OCs cultured with gentamicin showed significant loss of HCs. Addition of octreotide to gentamicin-treated OCs resulted in significantly decreased HC loss compared to in those treated with gentamicin only. However, addition of SH-6 and octreotide did not result in decreased hair cell loss. Scale bar  = 20 µm.

Treatment with SH-6, an inhibitor of Akt, alone (10 µM) did not result in HC damage. OC treated with SH-6 showed three orderly rows of outer HCs and a single row of inner HCs (Fig. 4). However, the protective effect of octretide on gentamicin-induced HC damage was reversed when SH-6 (10 µM) was added in addition to octreotide to the cell culture media (Fig. 5; ANOVA, p<0.01).

### Higher SST receptor-2 expression in SST recptor-1 knockout mice

**Figure 6:**
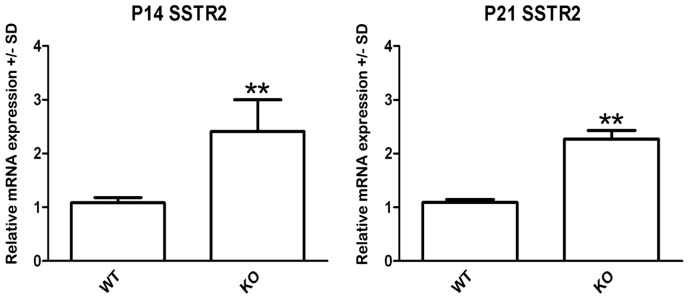
Cochlear gene expression of SST receptor-2 in the OC of postnatal day (P)14 and P21 wild-type (WT) and SST receptor-1 knockout (KO) mice. The relative distribution of SST receptor-2 mRNA expression in OC tissue from wild-type (WT) and SST receptor-1 KO of different postnatal ages was quantified by real-time PCR. GAPDH was used as an endogenous control. Gene expression levels are expressed as the mean (± one standard deviation) fold increase compared to the values obtained in OC explants from P14 and P21 WT and SST receptor-1 knockout mice. Data were obtained from five independent experiments, per condition in each experiment 5 OCs were used. **p<0.001 using Student's *t*-test.

At postnatal days 14 and 21, the mRNA gene expression of SST receptor-2 in OCs was two-fold higher in SST receptor-1 knockout mice compared to in their age-matched wild-type littermates (Fig. 6; unpaired Student's *t*-test, *p*<0.01).

### SST receptor-1 knockout is protective against gentamicin-induced HC damage in mouse OC explants

**Figure 7:**
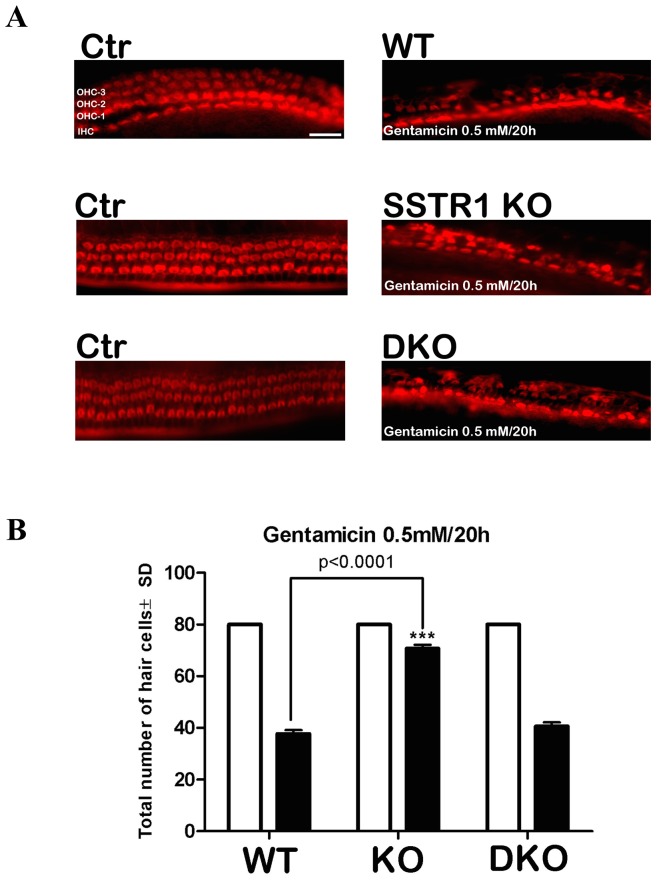
Quantitative analysis of surviving HCs. (a) Gentamicin-induced HC damage in the OCs of wild-type, SST receptor-1 knockout and SST receptor-1/SST receptor-2 double knockout mice. Photograph of phalloidin-labeled OC. The three outer HC (OHC) rows and a single inner HC (IHC) row can be seen in controls. OCs exposed to 0.5 mM gentamicin demonstrate HC loss. (b) Histograms show a significant difference in the number of surviving HCs in the OCs of SST receptor-1 knockout mice exposed to gentamicin compared to in the gentamicin-treated OCs of wild-type mice (*p*>0.0001). Histogram and bars represent mean ± one standard deviation. *n* = 15 for each experimental condition.

HC survival was increased in explants from SST receptor-1 knockout mice compared to in explants from wild-type and SST receptor-1/SST receptor-2 double-knockout mice after gentamicin (0.5 mM) exposure for 20 h. (Fig. 7; ANOVA, *p*<0.0001).

### Octreotide treatment results in increased SG neurite number

**Figure 8:**
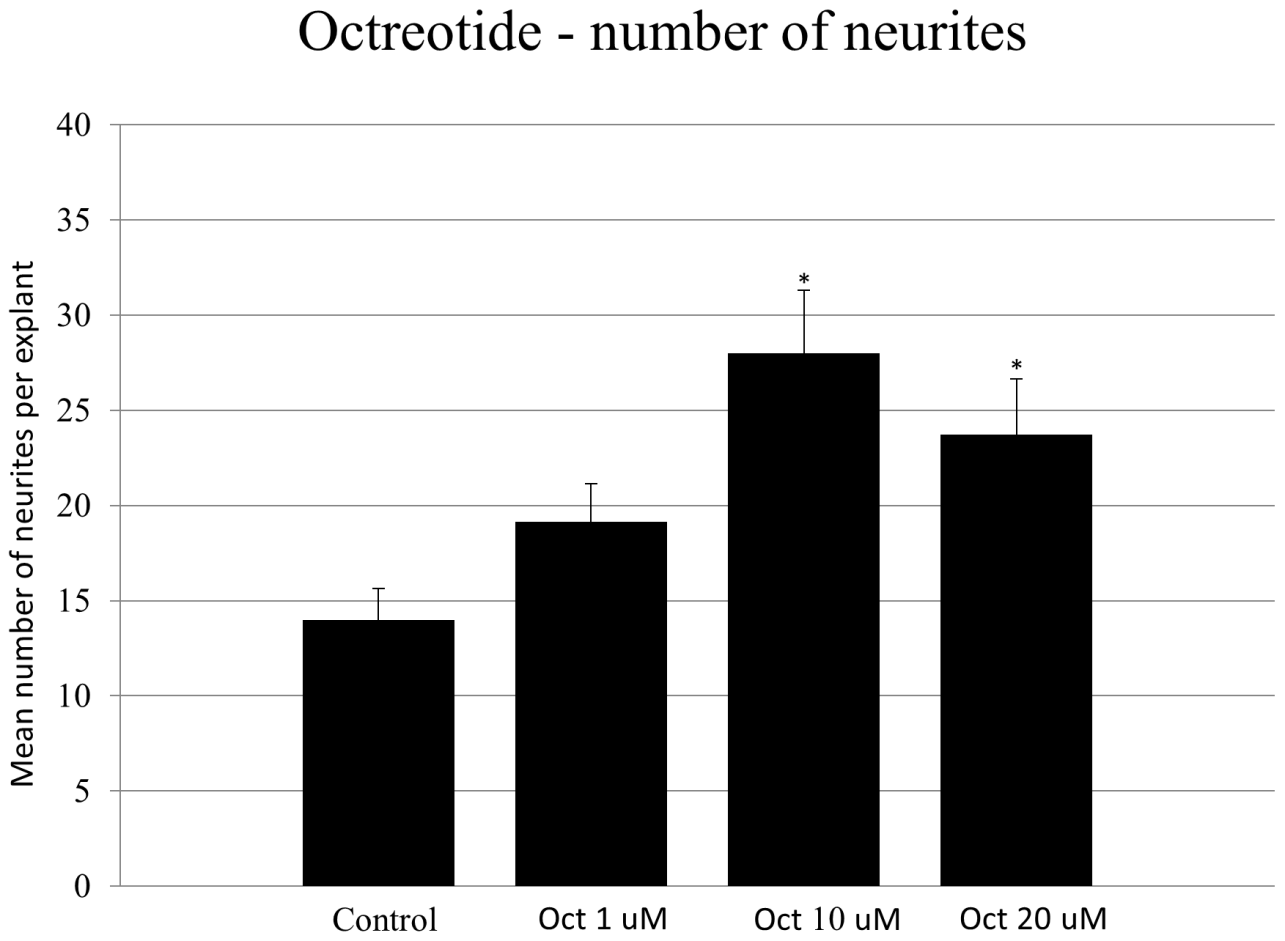
Average number of SG neurites observed from SG explants under the different experimental conditions. The number of neurites observed on control are compared to those seen with three different levels of octreotide (Oct). Lines represent one standard deviation. Asterisks denote statistical difference compared to control (p<0.05 for all conditions). *n* = 20 for each experimental condition.

**Figure 9:**
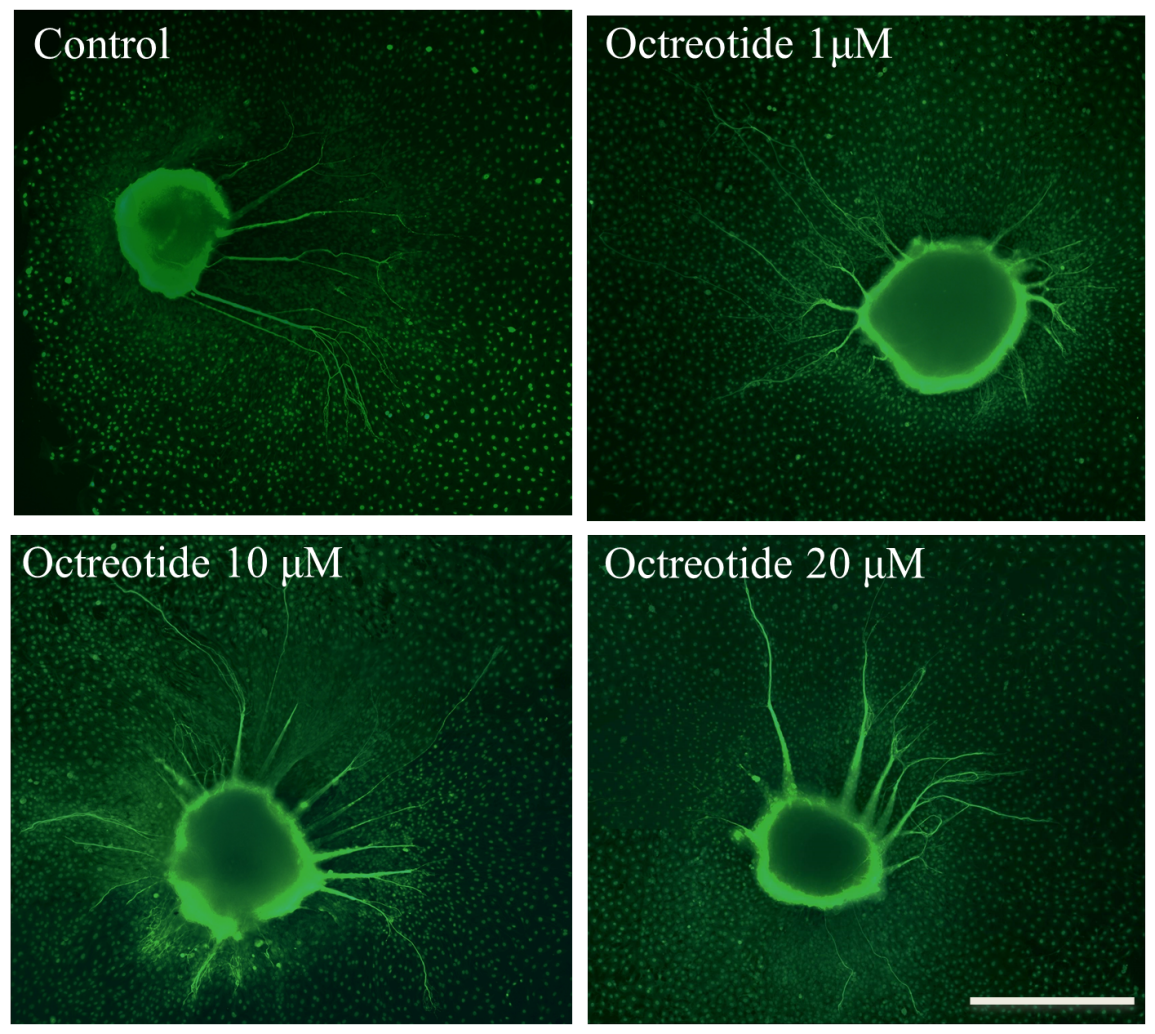
Effects of octreotide on SGNs *in vitro*. SGNs were treated with increasing concentrations of octreotide (1 µM, 10 µM and 20 µM, respectively) and compared to controls without octreotide treatment. Average number of SG neurites and average lenth of SG neurites observed from SG explants were analyzed. Representative SG explants stained with anti-200-kDa neurofilament antibody for each experimental condition are shown. Scale bar  = 300 µm.

Treatment of neonatal SG explants with the two highest concentrations of octreotide (10 or 20 µM) increased the number of neurites per SG explant compared to in controls (Fig. 8; ANOVA, *p*<0.05). Fig. 9 shows a representative image of SG explants treated with the different concentrations of octreotide compared to the control.

### Octreotide results in decreased length of SG neurites

**Figure 10:**
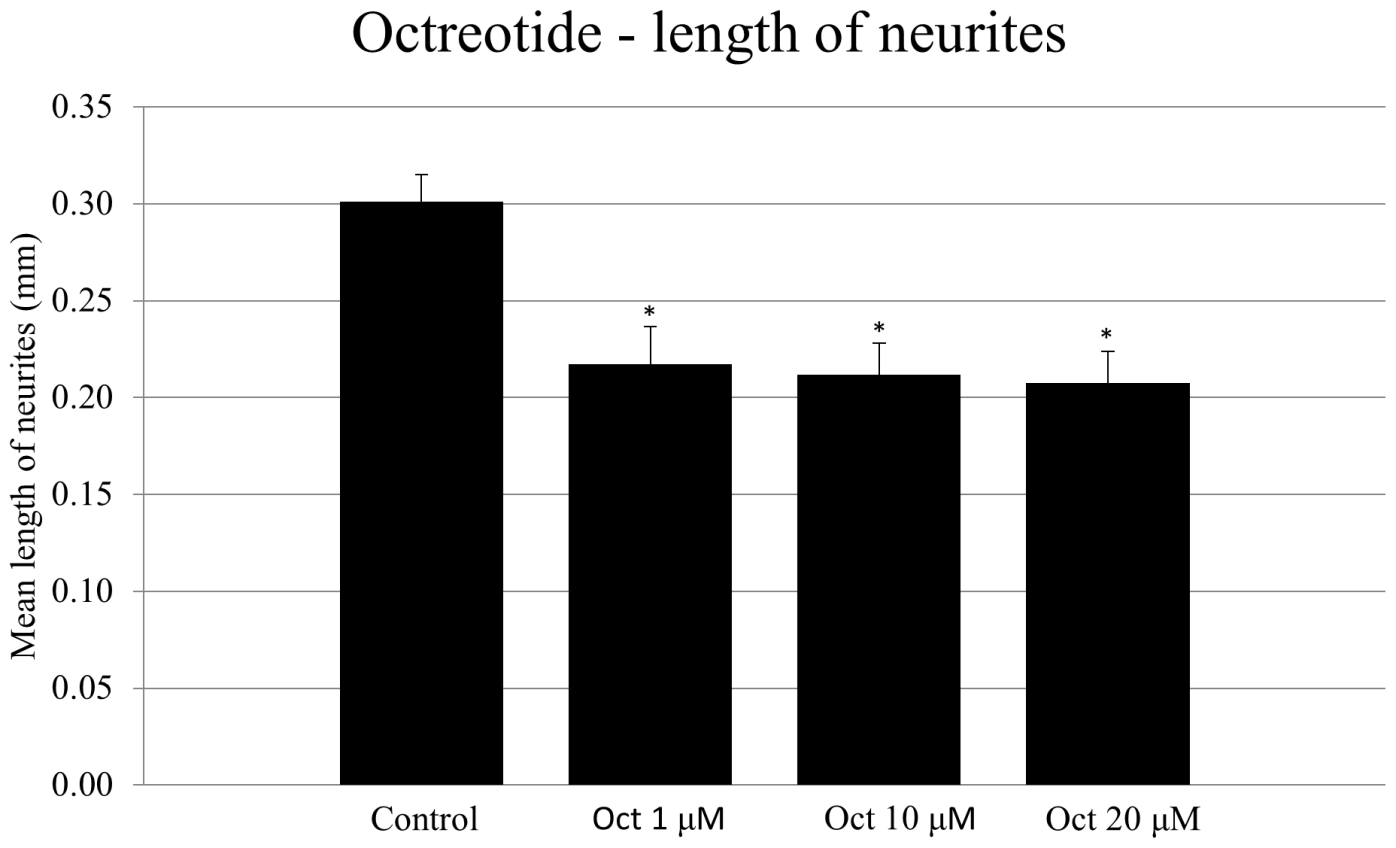
Average length of SG neurites observed on SG explants in the different experimental conditions. The lengths of neurites observed in the controls (media only) are compared to those seen with three different levels of octreotide (Oct). Lines represent one standard deviation. Asterisks denote statistical difference compared to control (p<0.05 for all experimental conditions). *n* = 20 for each experimental condition.

Treatment of neonatal SG explants with all concentrations of octreotide (1, 10, or 20 µM) significantly decreased the length of SG neurites compared to in controls (Fig. 10; ANOVA, *p*<0.05 for all conditions).

## Discussion

### Octreotide protects HCs from gentamicin-induced toxicity and activates Akt signaling *in vitro*

Interestingly, we found that octreotide significantly reduced HC loss in OC samples treated with gentamicin, as compared to in samples treated with gentamicin alone. Moreover, western blotting revealed that octreotide treatment increased Akt phosphorylation *in vitro and* inhibition of Akt by using the Akt inhibitor SH-6 reversed the protective effect of octreotide on gentamicin-induced HC loss *in vitro*. It should be noted that the cochlear explants for our tissue culture experiments were harvested from newborn animals since only newborn animals can be used for extended culture of inner ear HCs; adult HCs do not survive in culture [23]. A large number of studies have used aminoglycosides as HC death inducers, and the immature cochlea is an established *in vitro* model. However, since younger animals are more sensitive to ototoxins [1], [4], [6], some caution should be exercised in generalizing our present results to adults.

How can the protective effect of octreotide in gentamicin-induced HC loss be explained? To date, there are no previous reports of octreotide- or SST-induced intracellular events in the inner ear. However, there exist some data regarding SST-induced signaling in the visual system. The retina and inner ear share common characteristics: both arise from neuroepithelium, harbor sensitive sensory cells together with supporting cells, and display a complex and highly organized microarchitecture. Therefore, the molecular events involved in HC and SGN damage and death might share features similar to those involved in retinal cell damage and death [24], [25], [26]. In the retina, SST has pleiotropic effects and activates a variety of signaling mechanisms by its receptors, including PI3K, mitogen-activated kinases, and calcium channels [27].

Gentamicin exposure also activates pathways that promote HC survival, which supports the current opinion that cells exist in a finely tuned balance between survival and cell death [12], [28], [29]. Several survival pathways that operate in HCs have been defined, including the H-Ras/Raf/MEK/Erk pathway and the PI3K-Akt pathway [9]. Interestingly, Chung *et al*. demonstrated that PI3K/Akt mediates HC survival and opposes gentamicin toxicity in neonatal rat OC explants [12]. Moreover, we previously demonstrated that simvastatin protects HCs from gentamicin-induced toxicity and activates Akt signaling *in vitro* [30]. Therefore, we examined whether the observed protection from gentamicin by octreotide might be due to octreotide's influence on the PI3K/Akt pathway—and, in fact, our present results confirmed activation of Akt by octreotide. However, it is likely that other intracellular processes are involved, since somatostatin receptors modulate several intracellular signaling transduction pathways [16], [27]. It is also possible that somatostatin protects HCs from aminoglycoside toxicity-induced cell death through its ability to limit glutamate release or to inhibit glutamate excitotoxicity, as has been previously observed in the retina [31]. We must also consider the possibility that octreotide might interact physically with gentamicin. Although we have no direct evidence excluding physical interaction, this seems unlikely since the octreotide concentration was 10 to 50 times lower than the gentamicin concentration.

### Overexpression of SST receptor-2 protects HCs from gentamicin-induced toxicity *in vitro*

SST receptor-1 or SST receptor-2 deletion substantially alters the SST content in the retina [24], [25], [32]. In retinas from SST receptor KO mice, SST receptor-1 and SST receptor-2 expressions have been found to compensate for each other, such that SST receptor-1 loss results in increased expression of SST receptor-2 [32]. Notably, SST receptors also have a neuroprotective function in the retina. SST and its five receptors are expressed in the retina, predominantly in amacrine cells and bipolar cells [33]. Activation of SST receptor-2 by SST or its analogues reportedly protects retinal neurons against ischemia-induced damage [17], [26]. Additionally, studies in mice with genetic alterations of the somatostatinergic system have revealed that increased functional SST receptor-2 expression protects against retinal ischemia [34]. Therefore, SST receptor-2 analogs may have therapeutic benefits in retinal diseases, such as glaucoma or diabetic retinopathy.

Accordingly, our present results showed that SST receptor-1 KO mice exhibited up-regulation of SST receptor-2 in the OC compared to wild-type mice. We previously demonstrated that SST can dose-dependently protect HCs from aminoglycoside toxicity *in vitro* [18]. Interestingly, here we showed highly significant HC protection from aminoglycoside toxicity in the cochlea of SST receptor-1 knockout mice compared to in wild-type mice and SST receptor-1/SST receptor-2 double-knockout mice. The elevated level of SST receptor-2 might be responsible for this observed protection. However, up-regulation and interactions of other SST receptors must still be considered.

What mechanism might be responsible for the neuroprotective role of SST receptor-2 in the cochlea? Studies in mouse retinal explants have demonstrated that SST receptor-2 inhibits potassium-induced glutamate release [35]. By limiting the amount of glutamate available to glutamate receptors, SST and its analogs may exert neuroprotection against glutamate neurotoxicity, which characterizes many retinal diseases. Glutamate excitotoxicity appears to be mediated by caspase-3 activation, as shown in cerebrocortical neurons [36]. Glutamate excitotoxicity is also involved in HC damage and death in the cochlea [37]. Therefore, it is possible that SST protects HCs from aminoglycoside toxicity, either by limiting glutamate release or by mitigating the toxic action of excess glutamate on HCs. It is interesting to note that octreotide predominantly activates SST receptor-2 [38], [39]. The findings that octreotide offers a high degree of protection from gentamicin toxicity and that HC loss is reduced in SST receptor-1 mice that overexpress SST receptor-2 suggest an important function of SST receptor-2.

### Octreotide treatment results in increased SG neurite number and a slight decrease in SGN length *in vitro*

We found that octreotide protected HCs from gentamicin-induced HC damage, and we further demonstrated that octreotide induced neurite formation in neonatal rat SG explants. SST receptor-2 is the main receptor–pharmacological target mediating the effects of octreotide [38]. SST receptor-2 modulates several intracellular signaling transduction pathways in other systems, such as the PI3K-Akt and p38 pathways [16], [40]. SST receptor-2 reportedly mediates opposing proliferative effects through the activated p38 and activated Akt pathways in CHO-K1 cells *in vitro* [41]. While activation of p38 signaling generally promotes apoptosis [42], there are also several documented examples of survival enhancement by these pathways [43], [44]. We previously found that p38 and PI3K/Akt mediate BDNF-induced neurite formation in neonatal cochlear SG explants [45]. However, we did not assess the intracellular signaling pathways involved in octreotide-induced neurite formation in the present study. Therefore, we can only speculate that the p38 and PI3K/Akt intracellular signaling transduction pathways are involved in this process. Further studies are needed to elucidate the intracellular signaling transduction pathways involved in octreotide-induced SG neurite formation.

Interestingly, our data demonstrated a statistically significant decrease in average neurite length observed in octreotide-treated SG explants, indicating that octreotide mediates a negative effect on SG neuronal outgrowth. Tentler *et al*. showed that SST receptor-2 inhibits adenylate cyclase and, consequently, cAMP production in pituitary tumor GH4C1 cells, which ultimately results in decreased protein kinase A (PKA) activity [46]. Inhibition of PKA activity reportedly results in decreased in average neurite length and has no effect on neurite formation on neonatal rat SG explants. However, here we observed that inhibition of PI3K/Akt and Mek/Erk signaling resulted in increased neurite length, indicating that these pathways are involved in neurite elongation. Octreotide exposure may result in activation of intracellular signaling transduction pathways that have both positive and negative influences on neurite length, resulting in a total negative effect. Although this hypothesis may be too complex to be attractive without additional supporting data, it is at least consistent with our observations.

It should be noted that we could not distinguish between the dentrites and axons of SGNs, since we have not found markers that distinguish between the two in explants. Similarly, we could not distinguish between type I and type II SGN neurites, since peripherin labeling does not distinguish these two neuron classes in rat tissue cultures, due to up-regulation of peripherin type I neurons *in vitro* [47]. However, since 95% of SG neurons are type I cells, it seems likely that this class of neuron dominates our results.

## Conclusions

Our findings indicate that the SST analog octreotide highly protects HCs from gentamicin-induced toxicity and activates Akt signaling *in vitro*. Octreotide treatment also resulted in increased SG neurite number and a decrease in SGN length. Moreover, we discovered that SST receptor-1 knockout mice exhibited protection from aminoglycoside-induced HC loss compared to SST receptor-1/SST receptor-2 double-knockout and wild-type mice. SST receptor-2 was overexpressed in SST receptor-1 knockout mice, and might be responsible for the observed high degree of HC protection from gentamicin-induced HC loss. Our results suggest the somatostatinergic system in the inner ear to be a new and promising target in efforts to protect the mammalian inner ear from cell death.
